# Impact of dihydrogen bonding on lattice energies and sublimation enthalpies of crystalline [H_2_GaNH_2_]_3_, [H_2_BNH_2_]_3_ and [H_2_GeCH_2_]_3_†

**DOI:** 10.1039/c9ra07144j

**Published:** 2019-09-17

**Authors:** Wayne L. Gladfelter, Christopher J. Cramer

**Affiliations:** Department of Chemistry, University of Minnesota 207 Pleasant St., SE Minneapolis MN 55455 USA wlg@umn.edu; Department of Chemistry, Chemical Theory Center, Minnesota Supercomputing Institute, University of Minnesota 207 Pleasant St., SE Minneapolis MN 55455 USA

## Abstract

The lattice energies of [H_2_GaNH_2_]_3_, [H_2_BNH_2_]_3_ and [H_2_GeCH_2_]_3_ in their experimentally determined space groups, *P*2_1_/*m*, *Pmn*2_1_ and *Pbcm*, respectively, were calculated using density functional methods for periodic structures with the *ab initio* periodic code CRYSTAL17. Using the basis set pob-TZVP for all calculations, B3LYP including Grimme's D3 dispersion correction was found to reproduce experimental bond distances and angles most accurately. CRYSTAL17 was also used to optimize geometries and calculate energies of the molecular structures in the gas phase. While the chair conformation of the six-membered rings is found in all of the crystals, only [H_2_GeCH_2_]_3_ retains this as the preferred conformation in the gas phase. By contrast, a twist-boat conformation is preferred for both [H_2_GaNH_2_]_3_ and [H_2_BNH_2_]_3_ in the gas phase, and thus a correction for this change in conformation must be included in corresponding sublimation enthalpy calculations. In addition to the D3 dispersion correction, all lattice energies included a correction for basis set superposition error. The lattice energies for [H_2_GaNH_2_]_3_, [H_2_BNH_2_]_3_ and [H_2_GeCH_2_]_3_ were 153.5, 120.8 and 84.9 kJ mol^−1^, respectively. These values were used to calculate the sublimation enthalpies, which exhibited good agreement for the single case where an experimental measurement is available, namely [H_2_BNH_2_]_3_ (exp Δ*H*_sub_(298), 119 ± 12 kJ mol^−1^; calcd, 119.4 kJ mol^−1^). The energetic impact of the crystal structure was assessed by minimizing the structures of each molecule in each of the three space groups spanned by them experimentally and calculating their respective lattice energies. In every case, the experimentally observed space group was the one computed to be the most stable.

## Abstract 2

Calculated lattice energies and sublimation enthalpies provide quantitative measures of the importance of intermolecular dihydrogen bonds.

## Graphical abstract

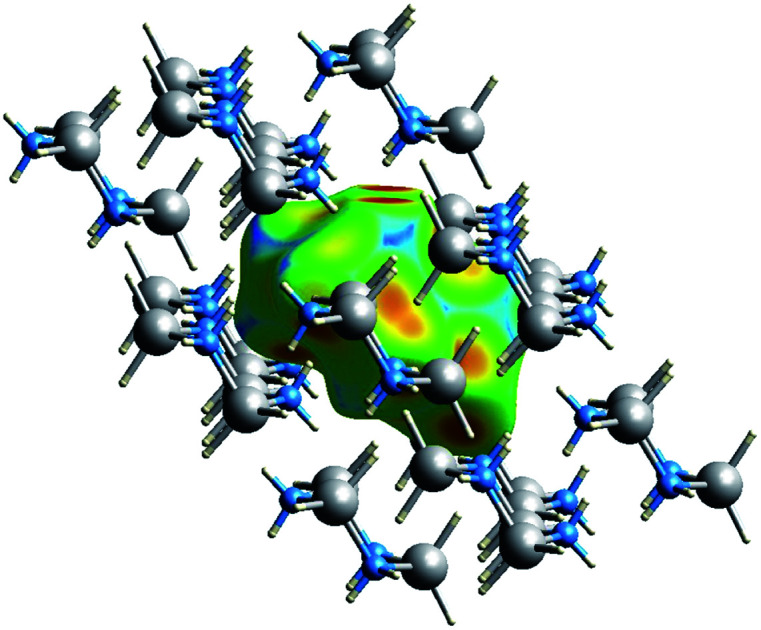

## Introduction

Volatility is a necessary property for molecules to function as precursors in chemical vapor deposition and related processes. In the case involving solid precursors, the heat of sublimation 
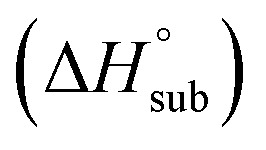
 is useful for predicting the equilibrium gas-phase concentration of a precursor. For molecular solids, lattice energy, the energy per molecule required to separate the molecules to gas-phase species, is the major contributor to the value of 
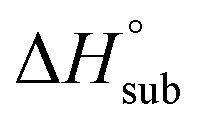
, and there has been much effort focused on using computational methods to predict 
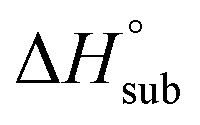
.^1–10^

Lattice energy depends on the strength of intermolecular bonds present in the crystalline phase and there has been great interest in structures exhibiting dihydrogen bonds. Ammonia–borane and related compounds, including [H_2_BNH_2_]_3_, exhibit intermolecular dihydrogen bonds and have been the focus of study due to their potential application in hydrogen storage systems.^11–13^ Numerous other main group metal compounds with hydrido ligands have been found to exhibit short intra- or intermolecular contacts with protic hydrogens.^1,11,14–22^ Dihydrogen bonds can also be important in the reactivity of the compounds.^11–13,16,17^ Structural studies of both cyclotrigallazane, [H_2_GaNH_2_]_3_,^16^ and cyclotriborazane, [H_2_BNH_2_]_3_,^22^ have revealed short intermolecular contacts between the hydridic hydrogens bound to the gallium or boron and the protic hydrogens bound to the nitrogens. A previous computational study of the gas phase dimers of [H_2_BNH_2_]_3_ and of [H_2_GaNH_2_]_3_ connected *via* dihydrogen bonds suggested a H⋯H bond energy of 13 kJ mol^−1^.^16^

**Fig. 1:**
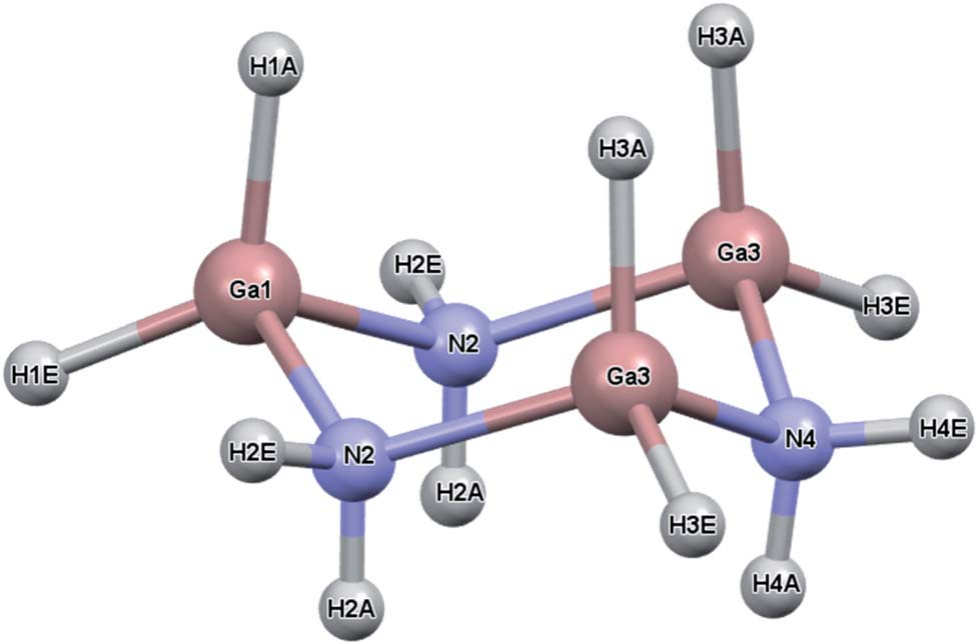
Atom labeling scheme for [H_2_GaNH_2_]_3_. Atoms 1 and 4 (Ga1 and N4 in the figure) along with their attached hydrogens lie on a crystallographic mirror plane. This is also true for [H_2_GeCH_2_]_3_ and [H_2_BNH_2_]_3_ where Ge and B atoms, respectively, replace the Ga atoms and C replaces the N in [H_2_GeCH_2_]_3_. The atom numbering is identical in all of the structures. The A and E labels on the hydrogens refer to the axial and equatorial positions, respectively.

While the previous study modeled the dihydrogen bond strength computationally based on the difference in energy between gas phase monomers and dimers, the current study includes all intermolecular interactions and reports heats of sublimation that in one case, [H_2_BNH_2_]_3_, can be compared to an experimental value.^23^ The current study expands on earlier work by calculating the lattice energy of crystalline [H_2_BNH_2_]_3_, [H_2_GaNH_2_]_3_ and [H_2_GeCH_2_]_3_. In the solid state, each of these molecules exist as a six-membered ring in a chair conformation. For convenience, the atomic labelling scheme was unified for all three molecules and is shown in Fig. 1 using [H_2_GaNH_2_]_3_ as an example. In their respective space groups, atoms 1 and 4 and their attached hydrogens of all three compounds reside on a crystallographic mirror plane. In this study, the lattice energy of each of the compounds in their native (experimentally determined) space group as well as in the space groups native to the other compounds was calculated. In each case the native space group was found to have the largest lattice energy, illustrating the manner in which the varying strengths of different intermolecular interactions can influence preferred packing arrangements.

## Computational methods

For calculations of crystalline [H_2_BNH_2_]_3_ (ref. 22) and [H_2_GeCH_2_]_3_ (ref. 24) the experimental crystal parameters and atomic coordinates obtained from single crystal X-ray diffraction results were used as the starting point. For [H_2_GaNH_2_]_3_ the crystal parameters and atomic coordinates resulting from Rietveld refinement of the neutron powder diffraction of the corresponding perdeutero compound were used.^16^ All calculations were made using the CRYSTAL17 code.^25^ The pob-TZVP basis set^26^ was used in all DFT calculations, and a shrinking factor of 4 was used to generate a grid of *k* points in reciprocal space. Four density functionals, B3LYP, PBE, PBE0 and M06-2x, were evaluated by comparing their results to the experimental structure of [H_2_GaNH_2_]_3_. For calculations using B3LYP, PBE and PBE0, Grimme's D3 dispersion correction,^27–29^ including Becke–Johnson damping,^30^ was employed by use of the keyword DFT-D3. Table 1 shows that the B3LYP and PBE functionals most closely reproduced the experimental results. B3LYP, which more closely reproduced the molecular structure, was chosen for all remaining calculations. Using the keyword MOLEBSSE invoked the counterpoise method to determine the basis set superposition error (BSSE).

**Table 1:** Comparison of experimental and calculated structures of [H_2_GaNH_2_]_3_ using different density functionals

	Method					
	XRD (EXP)	ND (EXP)	B3LYP	M06-2X	PBE	PBE0
Temp. (K)	106	298	0	0	0	0

**Lattice parameters**						
*a* (Å)	5.7615	5.7893	5.6471	5.6861	5.6607	5.6572
*b* (Å)	8.5079	8.5635	8.3703	8.3289	8.4648	8.3929
*c* (Å)	8.0848	8.1617	7.8564	7.7462	7.8960	7.8331
*β* (°)	110.843	111.038	110.347	110.095	110.846	110.987
Volume (Å^3^)	370.37	377.66	348.18	344.53	353.58	347.25
Density (g cm^−3^)	2.36	2.31^a^	2.49	2.52	2.45	2.50

**Average absolute errors**						
Cell axis dimensions (Å)			0.214	0.251	0.164	0.210
Bond lengths (Å)			0.026	0.037	0.087	0.115
Bond angles (°)			4.383	4.689	5.446	5.646

a Based on the formula [H_2_GaNH_2_]_3_.

Determination of the lattice energies required calculation of the energies of the isolated molecules in the chair conformation observed in the crystal structures. These calculations also used B3LYP and the same basis set used for the solid state calculations. For [H_2_GeCH_2_]_3_ the chair conformation was preferred in the gas phase, however, the twist-boat conformation was more stable for both [H_2_GaNH_2_]_3_ and [H_2_BNH_2_]_3_. The energy associated with this conformational change was included in the determination of the sublimation enthalpy. Vibrational frequency calculations were performed on both the gas phase and solid state structures in their native space groups using the keyword FREQCALC. From these calculations, zero point vibrational energies (ZPVE) and vibrational contributions to the sublimation enthalpy of each species at 298 K were determined.

Analysis of the Hirshfeld surfaces for each of the crystals used CrystalExplorer17.^31,32^

## Results and discussion

As reported previously the crystal and molecular structures of [H_2_GaNH_2_]_3_ and [D_2_GaND_2_]_3_ were solved by single crystal X-ray diffraction and Rietveld refinement of the powder neutron diffraction, respectively.^16^ For two reasons, the neutron diffraction results for [D_2_GaND_2_]_3_ were chosen as the source for comparison with the computational results. First, bond distances between heavy atoms and hydrogen determined using X-ray methods are known to be the shortened relative to those obtained using neutron methods. Because the calculated structures will report distances between nuclei positions, results from the neutron diffraction were considered more appropriate. Second, the twinning present in the single crystals affected the accuracy of the distances and angles in [H_2_GaNH_2_]_3_. Another difference between the two structural studies is the data collection temperature; 106 K for the X-ray diffraction experiment and 298 K for the neutron diffraction one. This led to a unit cell volume expansion of 1.97% for the higher temperature structure. As shown in Table 1, the calculated unit cell volumes at 0 K were 4–6% smaller regardless of the density functional used. At least part of this contraction can be assigned to the effect of temperature. In addition, part of the underestimation of the computed volumes could be ascribed to BSSE due to the finite basis set used for the calculations.^33^

The choice of density functional used for the calculations was based on how well it reproduced the experimental neutron diffraction results. One functional (PBE) and three hybrid functionals (PBE0, B3LYP and M06-2X) were tested using the same basis set (pob-TZVP). For calculations using the PBE, B3LYP and PBE0 functionals, Grimme's D3 dispersion correction was applied. In all calculations, both the atomic positional and unit cell parameters were allowed to refine to convergence within the chosen space group. Although the cell parameters (*a*, *b*, *c* and *β* for the native space *P*2_1_/*m* of [D_2_GaND_2_]_3_) were reproduced best using the PBE-D3 functional, B3LYP-D3 led to the smallest differences in bond lengths and angles of the molecular unit. The latter was chosen for all subsequent calculations. For purposes of comparison to the computational results, the density reported in Tables 1 and 2 for [D_2_GaND_2_]_3_ was calculated using the neutron diffraction cell volume for the protio formula. Tables 3 and 4 list the experimental and calculated metrical parameters for [H_2_GeCH_2_]_3_ and [H_2_BNH_2_]_3_, respectively.

**Table 2:** Selected metrical parameters of [H_2_GaNH_2_]_3_

	Method				
	XRD (EXP)	ND (EXP)	B3LYP	B3LYP	B3LYP
Temp. (K)	106	298	0	0	0
Crystal system	Monoclinic	Monoclinic	Monoclinic	Orthorhombic	Orthorhombic
Space group	*P*2_1_/*m*	*P*2_1_/*m*	*P*2_1_/*m*	*Pmn*2_1_	*Pbcm*
*Z*	2	2	2	2	4

**Lattice parameters**					
*a* (Å)	5.7615	5.7893	5.6471	8.4203	4.7423
*b* (Å)	8.5079	8.5635	8.3703	7.4080	13.7297
*c* (Å)	8.0848	8.1617	7.8564	5.6075	11.7629
*β* (°)	110.843	111.038	110.347		
Volume (Å^3^)	370.37	377.66	348.18	349.78	765.89
Density (g cm^−3^)	2.36	2.31^a^	2.49	2.48	2.26

**Average bond distances (Å)**					
Ga–N	1.978	1.976	1.995	1.995	1.993
Ga–HA		1.577	1.568	1.567	1.575
Ga–HE		1.537	1.570	1.571	1.562
N–HA		1.046	1.019	1.019	1.019
N–HE		1.026	1.018	1.018	1.018

**Close H–H nonbonded contacts (Å)**					
H2A–H3A		1.972	1.964	1.914	2.265
H2A–H1A					2.082
H1E–H4A					2.025

a Based on the formula [H_2_GaNH_2_]_3_.

**Table 3:** Selected metrical parameters of [H_2_GeCH_2_]_3_

	Method			
	XRD (EXP)	B3LYP	B3LYP	B3LYP
Temp. (K)	213	0	0	0
Crystal system	Orthorhombic	Orthorhombic	Monoclinic	Orthorhombic
Space group	*Pmn*2_1_	*Pmn*2_1_	*P*2_1_/*m*	*Pbcm*
*Z*	2	2	2	4

**Lattice parameters**				
*a* (Å)	8.663	8.431	5.847	5.068
*b* (Å)	7.783	7.365	8.336	14.019
*c* (Å)	6.124	5.836	7.833	10.730
*β* (°)			110.49	
Volume (Å^3^)	412.91	362.39	357.64	762.32
Density (g cm^−3^)	2.14	2.47	2.51	2.35

**Average bond distances (Å)**				
Ge–C	1.951	1.956	1.957	1.957
Ge–HA	1.572	1.531	1.536	1.537
Ge–HE	1.548	1.536	1.532	1.532
C–HA	1.107	1.088	1.088	1.089
C–HE	0.972	1.088	1.087	1.087

**Close H–H nonbonded contacts (Å)**				
H2A–H3A	2.200	2.101	2.143	
H2A–H1A				2.186

**Table 4:** Selected metrical parameters of [H_2_BNH_2_]_3_

	Method			
	XRD	B3LYP	B3LYP	B3LYP
Temp. (K)	180	0	0	0
Crystal system	Orthorhombic	Orthorhombic	Monoclinic	Orthorhombic
Space group	*Pbcm*	*Pbcm*	*P*2_1_/*m*	*Pmn*2_1_
*Z*	4	4	2	2

**Lattice parameters**				
*a* (Å)	4.383	4.248	5.004	7.358
*b* (Å)	12.193	11.914	7.343	6.635
*c* (Å)	11.180	10.917	7.225	5.025
*β* (°)			112.39	
Volume (cm^3^)	597.50	552.53	245.48	245.31
Density (g cm^−3^)	0.96	1.05	1.18	1.18

**Average bond distances (Å)**				
B–N	1.574	1.576	1.578	1.578
B–HA	1.133	1.208	1.201	1.203
B–HE	1.168	1.206	1.207	1.205
N–HA	0.863	1.020	1.021	1.021
N–HE	0.895	1.020	1.019	1.019

**Close H–H nonbonded contacts (**Å**)**				
H2A–H3A			1.882	1.912
H4E–H1E	2.275	2.022		
H4E–H1A	2.217	1.984		
H2E–H3A	2.259	2.009		
H2E–H3E	2.351	2.173		

**Fig. 2:**
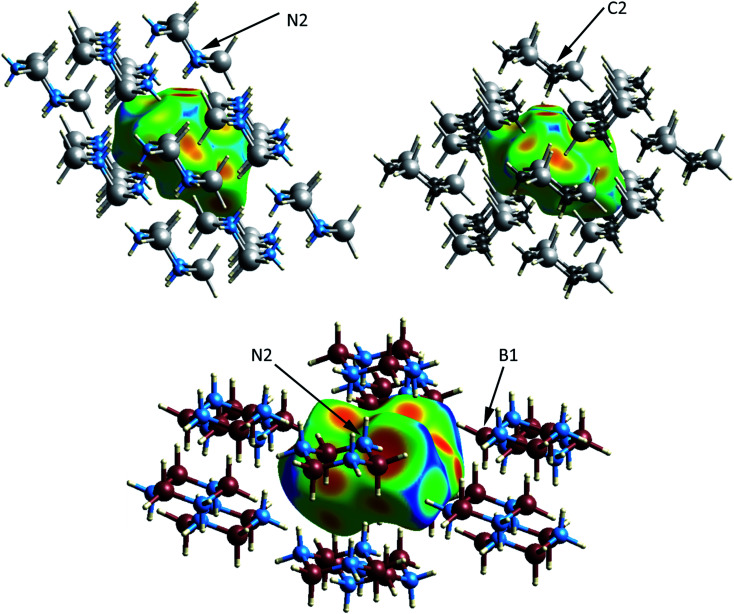
Hirshfeld surfaces of a molecule of [H_2_GaNH_2_]_2_ (upper left), [H_2_GeCH_2_]_3_ (upper right) and [H_2_BNH_2_]_3_ (lower) shown surrounded by 14 neighboring rings. The range of colors on the surface indicates distance of surrounding atoms to the surface with red representing the closer and blue the longer contacts.

The crystal and molecular structures of each of the compounds have been reported and compared elsewhere, and no further discussion of the molecular structure will be included here.^16,22,24^ An appreciation of the intermolecular interactions can be gleaned through the use of Hirshfeld surfaces as developed by Spackman and coworkers.^31,32^ Based on the calculated structures, the Hirshfeld surfaces are shown in Fig. 2. In each case the Hirshfeld surface is displayed for one molecule surrounded by 14 neighbors. The color code assesses the distance between the Hirshfeld surface and the neighboring atoms with red indicating the shortest distance, green intermediate and blue the longest. Despite their different space groups, the Hirshfeld surfaces of [H_2_GaNH_2_]_3_ and [H_2_GeCH_2_]_3_ and the corresponding contacts with neighboring molecules (as indicated by the red to yellow regions) are remarkably similar. In both cases all contacts result from Ga–H⋯H–N or Ge–H⋯H–C interactions. For both compounds the closest approach to the Hirshfeld surface can be seen at the top of the figure between the axial hydrogens attached to the nitrogen (labelled N2) in [H_2_GaNH_2_]_3_ and the carbon (C2) in [H_2_GeCH_2_]_3_.

**Fig. 3:**
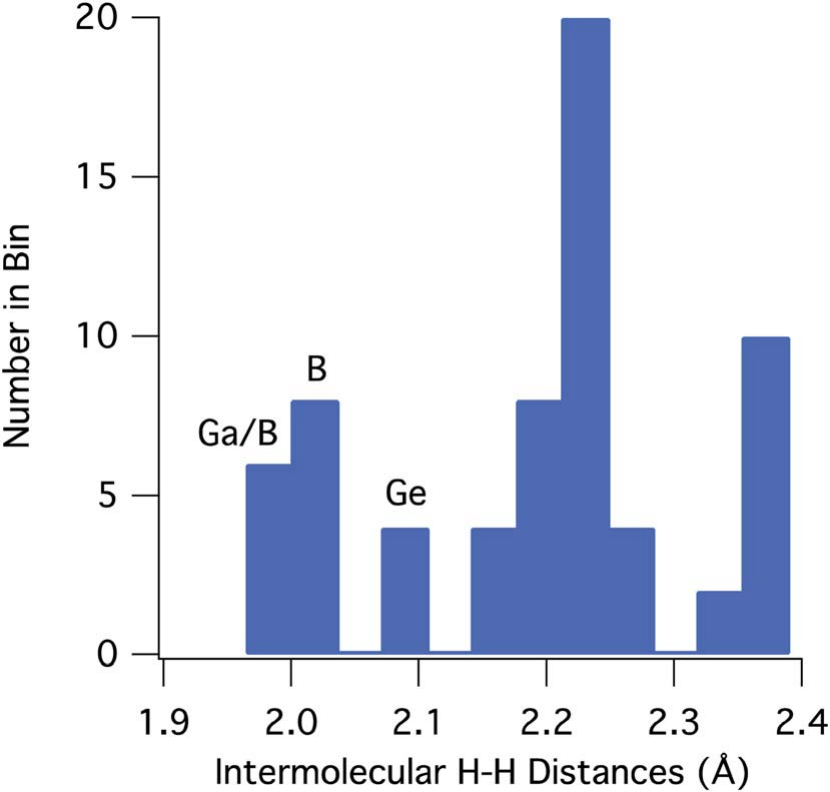
Histogram of the combined intermolecular H⋯H contacts for [H_2_GaNH_2_]_3_, [H_2_BNH_2_]_3_ and [H_2_GeCH_2_]_3_. The labels on the three shortest contact bins refer to the compounds contributing to that distance bin; Ga = [H_2_GaNH_2_]_3_, B = [H_2_BNH_2_]_3_, Ge = [H_2_GeCH_2_]_3_.

For [H_2_GaNH_2_]_3_ and [H_2_BNH_2_]_3_, there are 22 and 30 intermolecular H⋯H contacts between 1.9 and 2.4 Å, respectively. In this same range, [H_2_GeCH_2_]_3_ has 14 contacts among which only 4 shorter, symmetry equivalent contacts of 2.100 Å are found. All contacts below 2.4 Å occur between hydrides on a B, Ga or Ge and a hydrogen bound to a N or C. For the 66 H⋯H contacts in the three compounds, Fig. 3 shows a histogram of contact distances. Based on Bondi's van der Waal radius for hydrogen of 1.2 Å (ref. 34) previous reports suggest H⋯H distances below 2.4 Å constitute dihydrogen bonds. More recent studies of van der Waals radii suggest that a value of 1.1 Å is more appropriate for the hydrogen radius.^35,36^ Consistent with this shorter radius, the mode for the distribution in Fig. 3 includes contacts between 2.21 and 2.25 Å. All three compounds exhibit contacts shorter that 2.2 Å that can be reasonably considered as dihydrogen bonds. The shortest, and presumably the strongest, occur in [H_2_GaNH_2_]_3_ and [H_2_BNH_2_]_3_.

The number of H⋯H contacts per hydrogen differs in the three structures. In [H_2_BNH_2_]_3_ each of the axial hydrogens has three H⋯H contacts to neighboring molecules, whereas each of the equatorial hydrogens has two. The equatorial NH groups contact both hydrogens of an adjacent BH_2_ moiety to form an unsymmetrical, bifurcated dihydrogen bond. The equatorial hydrogen H4E that lies within the crystallographic mirror plane exhibits the shortest H⋯H contact of 1.984 Å (to H1A) and the second short contact (2.021 Å) is to H1E; both H1A and H1E are bonded to B1 (Fig. 2). Close inspection of the Hirshfeld surface in the region adjacent to B1 reveals two red spots corresponding to the bifurcated interaction with H4E. This interaction generates a chain of molecules connected by dihydrogen bonds parallel to the crystallographic *b*-axis in the *bc* plane. A second set of close contacts exists between the equatorial N–H (see N2 on Fig. 2) and the hydrides (H3A and H3E located within the Hirshfeld surface) attached to B3. The chain resulting from this interaction also lies in the *bc* plane but runs parallel to the *c*-axis. Longer H⋯H interactions connect molecules in the *ab* plane with the layers above and below. In contrast to [H_2_BNH_2_]_3_, most of the hydrogens in [H_2_GaNH_2_]_3_ and [H_2_GeCH_2_]_3_ exhibit two and one H⋯H contacts, respectively. The predominance of bifurcated dihydrogen bonds in cyclotriborazane compared to the complete lack of such interactions in cyclotrigallazane is likely attributable to the longer Ga–H bonds (1.57 Å) *vs.* the B–H distance of 1.21 Å and the wider H–Ga–H angle (119.7°) *vs.* H–B–H (111.6°). These metrical parameters would require the H–N proton to span a much larger distance between the two hydrogens on an HGaH group (2.71 Å) compared to 2.00 Å for an HBH group.

The Mulliken charges on each of the atoms (Table 5) confirm the hydridic nature of hydrogens attached to gallium, germanium and boron and the protic nature of those bound to nitrogen. The small positive charges on the carbon-bound hydrogens in [H_2_GeCH_2_]_3_ are undoubtedly a factor leading to the nonexistence of dihydrogen bonding in this compound.

**Table 5:** Mulliken charges for the compounds in their native space groups

Atom	[H_2_BNH_2_]_3_	[H_2_GaNH_2_]_3_	[H_2_GeCH_2_]_3_
	X = B, Y = N	X = Ga, Y = N	X = Ge, Y = C
X1	0.96	0.99	1.05
X3	0.95	1.00	1.03
Y2	−0.60	−0.89	−0.59
Y4	−0.58	−0.85	−0.59
H1A	−0.30	−0.27	−0.32
H1E	−0.31	−0.26	−0.28
H2A	0.12	0.22	0.07
H2E	0.13	0.20	0.07
H3A	−0.30	−0.28	−0.31
H3E	−0.30	−0.24	−0.27
H4A	0.13	0.18	0.07
H4E	0.11	0.22	0.07

### Calculated structures in non-native space groups

Considering the similar chair conformation of the molecular unit among these structures, we were curious to calculate each of the crystal and molecular structures in the alternative space groups. This was readily accomplished using the original atomic coordinates and lattice parameters as the starting point and changing the appropriate atoms for each calculation. All possibilities converged successfully. Table 2 compares the [H_2_GaNH_2_]_3_ experimental and calculated structures in both the native space group (*P*2_1_/*m*) and in the space groups for [H_2_GeCH_2_]_3_ (*Pmn*2_1_) and [H_2_BNH_2_]_3_ (*Pbcm*). The space group choice has little impact on the intramolecular distances and parameters, but it is interesting that the closest calculated intermolecular contact for [H_2_GaNH_2_]_3_ is slightly shorter (1.914 *vs.* 1.964 Å) in the non-native *Pmn*2_1_ space group. All calculated intermolecular contacts in *Pbcm* were longer than those found in *P*2_1_/*m* and *Pmn*2_1_. The intermolecular H⋯H contacts in [H_2_GeCH_2_]_3_ (Table 3) are longer than those calculated for [H_2_GaNH_2_]_3_ but the shortest contact occurs in the native space group. In the native space group for [H_2_BNH_2_]_3_ the intermolecular H⋯H contacts are longer than those calculated for either of the non-native space groups, which may reflect the impact of bifurcated bonding in determining the structure.

### Lattice energies

In an attempt to quantify the energetic impact of the crystal structure, lattice energies, *E*(lattice), were calculated for the three molecules in both their native and non-native space groups. Lattice energy is defined as the energy required to separate a mole of the crystalline solid into isolated gas phase molecules having the same conformation as in the solid state. In addition, the atom-centered calculations of CRYSTAL mandate correction for basis set superposition error, *E*(BSSE). In eqn (1), *E*(crystal) equals the crystal energy, *Z* equals the number of molecules in the unit cell, *E*(*C*_s_) equals the energy of a gaseous molecule having the same chair conformation (*C*_s_ point group) as observed in the solid. Density functional calculations for the gas phase molecules were conducted using the same functional and basis set (B3LYP-D3/pobTZVP) used for the solid-state structures.1
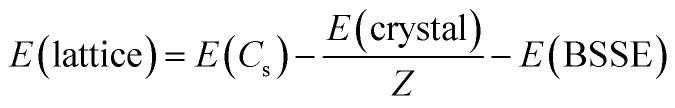


Table 6 lists each of the energies for the three compounds in each of the space groups. For each, the lattice energy calculated using CRYSTAL was largest for that compound's native space group. In each of the current compounds, the energy difference was less than 3 kJ mol^−1^ between *P*2_1_/*m* and *Pmn*2_1_. For [H_2_GaNH_2_]_3_ and [H_2_GeCH_2_]_3_, the lattice energy of the *Pbcm* space group was smaller by 13 to 19 kJ mol^−1^. For [H_2_BNH_2_]_3_, the *Pbcm* space was only 2.5 kJ mol^−1^ more stable that either of the others. Although the energy differences among the three space groups is small, there are no experimental results establishing the existence of polymorphs for these compounds.

**Table 6:** Lattice energies at 0 K (kJ mol^−1^)

Compound	*P*2_1_/*m*	*Pmn*2_1_	*Pbcm*
**[H**_**2**_ **GaNH**_**2**_ **]**_**3**_			
*E*(crystal)	−31 222 293.41	−31 222 290.63	−62 444 488.70
*Z*	2	2	4
*E*(*C*_s_)	−15 610 951.15	−15 610 951.15	−15 610 951.15
*E*(BSSE)	42.06	42.52	36.24
*E*(lattice)	**153.49**	151.65	134.79

**[H**_**2**_ **GeCH**_**2**_ **]**_**3**_			
*E*(crystal)	−33 356 028.40	−33 356 025.40	−66 711 984.15
*Z*	2	2	4
*E*(*C*_s_)	−16 677 867.31	−16 677 867.31	−16 677 867.31
*E*(BSSE)	64.7	60.52	60.11
*E*(lattice)	82.19	**84.87**	68.62

**[H**_**2**_ **BNH**_**2**_ **]**_**3**_			
*E*(crystal)	−1292 625.17	−1292 624.28	−2585 245.45
*Z*	2	2	4
*E*(*C*_s_)	−646157.94	−646157.94	−646157.94
*E*(BSSE)	36.33	35.91	32.66
*E*(lattice)	118.32	118.29	**120.77**

### Sublimation enthalpies

Eqn (2) was used to calculate the sublimation energy for each compound in their native space group (vibrational frequencies were not computed for the higher energy polymorphs). For [H_2_GaNH_2_]_3_ and [H_2_BNH_2_]_3_, the lowest energy conformation of the gas phase molecule differed from the molecular conformation in the solid state, thus requiring an additional term, Δ*E*(conf), in the calculation. For [H_2_GaNH_2_]_3_ and [H_2_BNH_2_]_3_ the twist-boat was preferred over the chair conformation by −16.8 and −5.0 kJ mol^−1^, respectively. These values compare to −10.9 and −3.8 kJ mol^−1^, respectively, based on the earlier calculations at the MP2/VDZ level of theory.^16^ For [H_2_GeCH_2_]_3_, the chair was calculated to be more stable than the twist-boat conformation by 4.4 kJ mol^−1^, and thus no conformation correction was needed.

2 Δ*H*_sub_(*T*) = *E*(lattice) + Δ*E*_conf_ + Δ*E*_ZPVE_ + Δ*E*_vib_(*T*) + 4*RT*

The next two terms in eqn (2) are the difference in zero point vibrational energy between the crystalline and gaseous states, Δ*E*_ZPVE_, and the difference in the vibrational contributions at temperature *T* of the crystalline and gaseous states, Δ*E*_vib_(*T*). The 4*RT* term accounts for the rotational, translational and *pV* work contributions to the energy of the gaseous product. Table 7 summarizes all contributions and the final Δ*H*_sub_ for each molecule at 298 K.

**Table 7:** Enthalpies of sublimation at 298 K. All energies have units of kJ mol^−1^

Compound	[H_2_GaNH_2_]_3_	[H_2_GeCH_2_]_3_	[H_2_BNH_2_]_3_
Space group	*P*2_1_/*m*	*Pmn*2_1_	*Pbcm*
*Z*	2	2	4
*T* (K)	298.15	298.15	298.15
*E*(lattice)	153.49	84.87	120.77
Δ*E*(conf)	−16.83	0.00	−4.95
ZPVE(crystal)/*Z*	341.06	347.41	427.74
ZPVE(gas)	334.31	343.04	422.61
*E*_vib_(crystal)/*Z* at *T*	29.76	26.83	18.51
*E*_vib_(gas) at *T*	30.11	26.29	17.89
4*RT*(gas)	9.92	9.92	9.92
Δ*H*_sub_(*T*, calcd)	140.18	89.89	119.43
Δ*H*_sub_(*T*, exp)	na	na	119 ± 12

Experimentally, neither [H_2_BNH_2_]_3_ nor [H_2_GaNH_2_]_3_ exhibited a detectable melting point prior to decomposing at 150 °C.^16,23^ Both sublimed under high vacuum above temperatures of 80–90 °C, whereas [H_2_GeCH_2_]_3_ had a melting point of −14 °C and was purified by distillation at 65 °C under reduced pressure (11 mbar).^24^ Using a Knudson cell, Shore and coworkers measured the vapor pressure of [H_2_BNH_2_]_3_ in the range from 47.5 to 75.5 °C to establish its heat of sublimation as 105 ± 13 kJ mol^−1^.^23^ Using the center of their temperature range, the Δ*H*_sub_ was converted to the value at 298.15 K using the method described by Chickos and Acree and the calculated heat capacities for the crystalline and molecular states.^37^ The agreement was good between the experimental (119 ± 12 kJ mol^−1^) and calculated (119.4 kJ mol^−1^) values.

## Conclusions

The crystal and molecular structures of [H_2_BNH_2_]_3_, [H_2_GaNH_2_]_3_ and [H_2_GeCH_2_]_3_ were successfully modeled using periodic DFT calculations in their native space groups of (*Pbcm*, *P*2_1_/*m* and *Pmn*2_1_, respectively). The calculated structures provided a basis for a more uniform comparisons among the structures. In each compound, all intermolecular H⋯H contacts occur between hydridic and protic hydrogens, and the majority of the H⋯H distances occur at or slightly above the expected van der Waals distance (2.2 Å). Both [H_2_BNH_2_]_3_ and [H_2_GaNH_2_]_3_ exhibit several contacts that are ∼0.2 Å shorter than the van der Waals contact distance, which places them in the range of typical dihydrogen bonds. The shortest H⋯H contacts in [H_2_GeCH_2_]_3_ (2.1 Å) are intermediate between the van der Waals and dihydrogen bonding distances. Comparison of the crystal energies to the energy of the gas phase molecules having the same chair conformation found in the solid state yielded lattice energies of 120.77, 153.49 and 84.87 kJ mol^−1^, respectively. For comparison, the crystal and molecular structure of each compound were also calculated in the two non-native space groups (*e.g. P*2_1_/*m* and *Pmn*2_1_ for [H_2_BNH_2_]_3_). In each case the largest lattice energy corresponded to the experimentally observed (native) space group. For the gas phase molecules and the compounds in their native space group, vibrational frequency calculations allowed calculation of their sublimation enthalpies. For [H_2_BNH_2_]_3_ and [H_2_GaNH_2_]_3_ the sublimation enthalpy calculation included a contribution associated with the conformational difference between the solid state and gas phase conformations. Good agreement was found between the calculated sublimation energy of [H_2_BNH_2_]_3_ (119.4 kJ mol^−1^) and the published experimental value (119 ± 12 kJ mol^−1^).

## Conflicts of interest

There are no conflicts to declare.

## Supplementary Material

RA-009-C9RA07144J-s001
