# Supplementary material for: Impact of dihydrogen bonding on lattice energies and sublimation enthalpies of crystalline [H2GaNH2]3, [H2BNH2]3 and [H2GeCH2]3
Source: RSC Adv. 2019 Sep 17;9(50):29448–55. doi: 10.1039/c9ra07144j (PMC9071838; doi:10.1039/c9ra07144j)
Supplement: RA-009-C9RA07144J-s001 [file RA-009-C9RA07144J-s001.pdf]

Electronic Supplementary Material (ESI) for RSC Advances.
This journal is © The Royal Society of Chemistry 2019

Electronic supplementary information for

Impact of Dihydrogen Bonding on Lattice Energies and Sublimation Enthalpies of Crystalline $[H_2GaNH_2]_3$, $[H_2BNH_2]_3$ and $[H_2GeCH_2]_3$†

Wayne L. Gladfelter*[a] and Christopher J. Cramer[b]

The calculated cell parameters and atomic coordinates for each compound in the space groups, *P2₁/m*, *Pmn2₁* and *Pbcm* are listed in the format of a crystallographic information file(cif).

```
data_cyclotriborane_P21/m
_audit_creation_method        'generated by CrystalMaker 9.2.7'
_publ_section_comment
;
(Imported from a VASP structure file)

H 48   B 12   N 12
;
_cell_length_a               5.0044(0)
_cell_length_b               7.3429(0)
_cell_length_c               7.2251(0)
_cell_angle_alpha            90.0000(0)
_cell_angle_beta             112.3930(0)
_cell_angle_gamma            90.0000(0)

_symmetry_space_group_name_H-M     'P 21/m'
_symmetry_Int_Tables_number        11
_symmetry_cell_setting             monoclinic
loop_
_symmetry_equiv_pos_as_xyz
'+x,+y,+z'
'-x,1/2+y,-z'
'-x,-y,-z'
'+x,1/2-y,+z'

loop_
_atom_site_label
_atom_site_type_symbol
_atom_site_occupancy
_atom_site_fract_x
```

```
_atom_site_fract_y
_atom_site_fract_z
   B1    B  1.0000     -0.7681    0.2500    0.4681
   B3    B  1.0000     -0.1696    0.4302    0.1716
  H1A    H  1.0000     -0.9123    0.2500   -0.4317
  H1E    H  1.0000     -0.5129    0.2500   -0.4292
  H2A    H  1.0000     -0.7070    0.4348    0.2564
  H2E    H  1.0000     -0.8124   -0.4640    0.4155
  H3A    H  1.0000     -0.3283    0.4327    0.2613
  H3E    H  1.0000     -0.2037   -0.4373    0.0666
  H4A    H  1.0000     -0.1095    0.2500   -0.0445
  H4E    H  1.0000     -0.4393    0.2500   -0.0540
   N2    N  1.0000     -0.8475    0.4228    0.3277
   N4    N  1.0000     -0.2287    0.2500    0.0429


data_cyclotriborazane_Pbcm
_audit_creation_method        'generated by CrystalMaker 9.2.7'
_publ_section_comment
;
(Imported from a VASP structure file)

H 96   B 24   N 24
;
_cell_length_a                 4.2481(0)
_cell_length_b                11.9138(0)
_cell_length_c                10.9173(0)
_cell_angle_alpha              90.0000(0)
_cell_angle_beta               90.0000(0)
_cell_angle_gamma              90.0000(0)

_symmetry_space_group_name_H-M     'P b c m'
_symmetry_Int_Tables_number        57
_symmetry_cell_setting           orthorhombic
loop_
_symmetry_equiv_pos_as_xyz
'+x,+y,+z'
'-x,-y,1/2+z'
'-x,1/2+y,1/2-z'
'+x,1/2-y,-z'
'-x,-y,-z'
'+x,+y,1/2-z'
'+x,1/2-y,1/2+z'
'-x,1/2+y,+z'
```

```
loop_
_atom_site_label
_atom_site_type_symbol
_atom_site_occupancy
_atom_site_fract_x
_atom_site_fract_y
_atom_site_fract_z
   B1    B  1.0000    -0.1446   -0.3857    0.2500
   B3    B  1.0000    -0.1469    0.4214    0.3716
  H1A    H  1.0000    -0.8601   -0.3833    0.2500
  H1E    H  1.0000    -0.2556   -0.2927    0.2500
  H2A    H  1.0000    -0.4968   -0.4476    0.3739
  H2E    H  1.0000    -0.1747   -0.4125    0.4427
  H3A    H  1.0000    -0.8628    0.4196    0.3740
  H3E    H  1.0000    -0.2580    0.3744    0.4596
  H4A    H  1.0000    -0.4966    0.3528    0.2500
  H4E    H  1.0000    -0.1704    0.2829    0.2500
   N2    N  1.0000    -0.2578   -0.4521    0.3665
   N4    N  1.0000    -0.2581    0.3626    0.2500


data_cyclotriborazane_Pmn21
_audit_creation_method        'generated by CrystalMaker 9.2.7'
_publ_section_comment
;
(Imported from a VASP structure file)

H 48   B 12   N 12
;
_cell_length_a                 7.3582(0)
_cell_length_b                 6.6349(0)
_cell_length_c                 5.0246(0)
_cell_angle_alpha              90.0000(0)
_cell_angle_beta               90.0000(0)
_cell_angle_gamma              90.0000(0)

_symmetry_space_group_name_H-M     'P m n 21'
_symmetry_Int_Tables_number        31
_symmetry_cell_setting             orthorhombic
loop_
_symmetry_equiv_pos_as_xyz
'+x,+y,+z'
'1/2-x,-y,1/2+z'
```

```
'1/2+x,-y,1/2+z'
'-x,+y,+z'

loop_
_atom_site_label
_atom_site_type_symbol
_atom_site_occupancy
_atom_site_fract_x
_atom_site_fract_y
_atom_site_fract_z
   B1    B  1.0000    -0.5000    0.0357    0.4083
   B3    B  1.0000    -0.3204    0.3267    0.1527
  H1A    H  1.0000    -0.5000   -0.0750    0.2183
  H1E    H  1.0000    -0.5000   -0.0574   -0.3858
  H2A    H  1.0000    -0.3153    0.2539   -0.4294
  H2E    H  1.0000    -0.2148    0.0874    0.3877
  H3A    H  1.0000    -0.3192    0.2310   -0.0501
  H3E    H  1.0000    -0.1878    0.4329    0.1693
  H4A    H  1.0000    -0.5000   -0.4449    0.3149
  H4E    H  1.0000    -0.5000   -0.4555   -0.0086
   N2    N  1.0000    -0.3274    0.1768    0.3956
   N4    N  1.0000    -0.5000    0.4572    0.1582


data_cyclotrigallazane_P21/m
_audit_creation_method        'generated by CrystalMaker 9.2.7'
_publ_section_comment
;
(Imported from a VASP structure file)

H 24   N 6   Ga 6
;
_cell_length_a                5.6471(0)
_cell_length_b                8.3703(0)
_cell_length_c                7.8563(0)
_cell_angle_alpha               90.0000(0)
_cell_angle_beta              110.3470(0)
_cell_angle_gamma               90.0000(0)

_symmetry_space_group_name_H-M     'P 21/m'
_symmetry_Int_Tables_number        11
_symmetry_cell_setting           monoclinic
loop_
_symmetry_equiv_pos_as_xyz
```

'+x,+y,+z'
'-x,1/2+y,-z'
'-x,-y,-z'
'+x,1/2-y,+z'

loop_
_atom_site_label
_atom_site_type_symbol
_atom_site_occupancy
_atom_site_fract_x
_atom_site_fract_y
_atom_site_fract_z
Ga1 Ga 1.0000 0.7629 0.2500 0.5010
Ga3 Ga 1.0000 0.3139 0.4498 0.1701
H1A H 1.0000 0.5679 0.2500 0.6028
H1E H 1.0000 0.0538 0.2500 0.6122
H2A H 1.0000 0.7891 0.4319 0.2516
H2E H 1.0000 0.7226 0.5362 0.4021
H3A H 1.0000 0.1536 0.4301 0.2961
H3E H 1.0000 0.2670 0.5979 0.0397
H4A H 1.0000 0.4115 0.2500 0.9629
H4E H 1.0000 0.1096 0.2500 0.9229
N2 N 1.0000 0.6753 0.4337 0.3278
N4 N 1.0000 0.2802 0.2500 0.0249

data_cyclotrigallazane_pbcm2
_audit_creation_method 'generated by CrystalMaker 9.2.7'
_publ_section_comment
;
(Imported from a VASP structure file)

H 96 N 24 Ga 24
;
_cell_length_a 4.7423(0)
_cell_length_b 13.7297(0)
_cell_length_c 11.7629(0)
_cell_angle_alpha 90.0000(0)
_cell_angle_beta 90.0000(0)
_cell_angle_gamma 90.0000(0)

_symmetry_space_group_name_H-M 'P b c m'
_symmetry_Int_Tables_number 57
_symmetry_cell_setting orthorhombic

```
loop_
_symmetry_equiv_pos_as_xyz
'+x,+y,+z'
'-x,-y,1/2+z'
'-x,1/2+y,1/2-z'
'+x,1/2-y,-z'
'-x,-y,-z'
'+x,+y,1/2-z'
'+x,1/2-y,1/2+z'
'-x,1/2+y,+z'

loop_
_atom_site_label
_atom_site_type_symbol
_atom_site_occupancy
_atom_site_fract_x
_atom_site_fract_y
_atom_site_fract_z
  Ga1   Ga 1.0000    -0.1353   -0.3920    0.2500
  Ga3   Ga 1.0000    -0.1575    0.3964    0.3935
  H1A    H  1.0000    -0.8078   -0.4097    0.2500
  H1E    H  1.0000    -0.2373   -0.2838    0.2500
  H2A    H  1.0000    -0.5182   -0.4697    0.3632
  H2E    H  1.0000    -0.2827   -0.4312    0.4512
  H3A    H  1.0000    -0.8268    0.4056    0.3826
  H3E    H  1.0000    -0.2816    0.3473   -0.4977
  H4A    H  1.0000    -0.5133    0.3359    0.2500
  H4E    H  1.0000    -0.2414    0.2656    0.2500
   N2    N  1.0000    -0.3059   -0.4679    0.3765
   N4    N  1.0000    -0.2984    0.3371    0.2500


data_cyclotrigallazane_Pmn21
_audit_creation_method        'generated by CrystalMaker 9.2.7'
_publ_section_comment
;
(Imported from a VASP structure file)

H 48   N 12   Ga 12
;
_cell_length_a               8.4203(0)
_cell_length_b               7.4080(0)
_cell_length_c               5.6075(0)
_cell_angle_alpha              90.0000(0)
```

```
_cell_angle_beta              90.0000(0)
_cell_angle_gamma             90.0000(0)

_symmetry_space_group_name_H-M     'P m n 21'
_symmetry_Int_Tables_number        31
_symmetry_cell_setting            orthorhombic
loop_
_symmetry_equiv_pos_as_xyz
'+x,+y,+z'
'1/2-x,-y,1/2+z'
'1/2+x,-y,1/2+z'
'-x,+y,+z'

loop_
_atom_site_label
_atom_site_type_symbol
_atom_site_occupancy
_atom_site_fract_x
_atom_site_fract_y
_atom_site_fract_z
  Ga1    Ga 1.0000    -0.5000   -0.0034    0.4113
  Ga3    Ga 1.0000    -0.3001    0.3326    0.1332
  H1A    H  1.0000    -0.5000   -0.1035    0.1649
  H1E    H  1.0000    -0.5000   -0.1130   -0.3487
  H2A    H  1.0000    -0.3210    0.2416   -0.4305
  H2E    H  1.0000    -0.2160    0.0965    0.4237
  H3A    H  1.0000    -0.3148    0.2090   -0.0921
  H3E    H  1.0000    -0.1541    0.4627    0.1619
  H4A    H  1.0000    -0.5000   -0.4547    0.3178
  H4E    H  1.0000    -0.5000   -0.4293    0.0298
  N2     N  1.0000    -0.3182    0.1700    0.4143
  N4     N  1.0000    -0.5000    0.4759    0.1611


data_1,3,5-trigermacyclohexane_P21/m
_audit_creation_method        'generated by CrystalMaker 9.2.7'
_publ_section_comment
;
(Imported from a VASP structure file)

H 48   C 12   Ge 12
;
_cell_length_a                5.8469(0)
_cell_length_b                8.3360(0)
```

```
_cell_length_c                 7.8333(0)
_cell_angle_alpha              90.0000(0)
_cell_angle_beta               110.4870(0)
_cell_angle_gamma              90.0000(0)

_symmetry_space_group_name_H-M     'P 21/m'
_symmetry_Int_Tables_number        11
_symmetry_cell_setting             monoclinic
loop_
_symmetry_equiv_pos_as_xyz
'+x,+y,+z'
'-x,1/2+y,-z'
'-x,-y,-z'
'+x,1/2-y,+z'

loop_
_atom_site_label
_atom_site_type_symbol
_atom_site_occupancy
_atom_site_fract_x
_atom_site_fract_y
_atom_site_fract_z
   C2    C  1.0000    -0.8448    0.4454    0.3299
   C4    C  1.0000    -0.2350    0.2500    0.0043
  Ge1    Ge 1.0000    -0.7357    0.2500    0.4748
  Ge3    Ge 1.0000    -0.1817    0.4404    0.1594
  H1A    H  1.0000    -0.8443    0.2500   -0.3727
  H1E    H  1.0000    -0.4575    0.2500   -0.4364
  H2A    H  1.0000    -0.7256    0.4672    0.2523
  H2E    H  1.0000    -0.8225   -0.4535    0.4221
  H3A    H  1.0000    -0.3572    0.4291    0.2660
  H3E    H  1.0000    -0.2408   -0.4042    0.0473
  H4A    H  1.0000    -0.1109    0.2500   -0.0717
  H4E    H  1.0000    -0.4204    0.2500   -0.0936


data_1,3,5-trigermacyclohexane_pbcm
_audit_creation_method        'generated by CrystalMaker 9.2.7'
_publ_section_comment
;
(Imported from a VASP structure file)

H 96   C 24   Ge 24
;
```

```
_cell_length_a                5.0677(0)
_cell_length_b                14.0192(0)
_cell_length_c                10.7301(0)
_cell_angle_alpha             90.0000(0)
_cell_angle_beta              90.0000(0)
_cell_angle_gamma             90.0000(0)

_symmetry_space_group_name_H-M     'P b c m'
_symmetry_Int_Tables_number        57
_symmetry_cell_setting             orthorhombic
loop_
_symmetry_equiv_pos_as_xyz
'+x,+y,+z'
'-x,-y,1/2+z'
'-x,1/2+y,1/2-z'
'+x,1/2-y,-z'
'-x,-y,-z'
'+x,+y,1/2-z'
'+x,1/2-y,1/2+z'
'-x,1/2+y,+z'

loop_
_atom_site_label
_atom_site_type_symbol
_atom_site_occupancy
_atom_site_fract_x
_atom_site_fract_y
_atom_site_fract_z
   C2    C  1.0000     -0.2598   -0.4934    0.3939
   C4    C  1.0000     -0.4056    0.3142    0.2500
  Ge1    Ge 1.0000     -0.0824   -0.4376    0.2500
  Ge3    Ge 1.0000     -0.2461    0.3669    0.4014
  H1A    H  1.0000     -0.7885   -0.4648    0.2500
  H1E    H  1.0000     -0.1028   -0.3286    0.2500
  H2A    H  1.0000     -0.4667   -0.4739    0.3851
  H2E    H  1.0000     -0.1901   -0.4628    0.4807
  H3A    H  1.0000     -0.9583    0.3330    0.4107
  H3E    H  1.0000     -0.3919    0.3345   -0.4810
  H4A    H  1.0000     -0.6158    0.3308    0.2500
  H4E    H  1.0000     -0.3866    0.2369    0.2500


data_1,3,5-trigermacyclohexane_Pmn21
_audit_creation_method        'generated by CrystalMaker 9.2.7'
```

```
_publ_section_comment
;
(Imported from a VASP structure file)

H 48   C 12   Ge 12
;
_cell_length_a                8.4314(0)
_cell_length_b                7.3651(0)
_cell_length_c                5.8356(0)
_cell_angle_alpha             90.0000(0)
_cell_angle_beta              90.0000(0)
_cell_angle_gamma             90.0000(0)

_symmetry_space_group_name_H-M     'P m n 21'
_symmetry_Int_Tables_number        31
_symmetry_cell_setting             orthorhombic
loop_
_symmetry_equiv_pos_as_xyz
'+x,+y,+z'
'1/2-x,-y,1/2+z'
'1/2+x,-y,1/2+z'
'-x,+y,+z'

loop_
_atom_site_label
_atom_site_type_symbol
_atom_site_occupancy
_atom_site_fract_x
_atom_site_fract_y
_atom_site_fract_z
   C2    C  1.0000    -0.3102    0.1581    0.4029
   C4    C  1.0000    -0.5000    0.4920    0.1768
  Ge1    Ge 1.0000    -0.5000    0.0052    0.3944
  Ge3    Ge 1.0000    -0.3091    0.3419    0.1608
  H1A    H  1.0000    -0.5000   -0.1049    0.1728
  H1E    H  1.0000    -0.5000   -0.1275   -0.4022
  H2A    H  1.0000    -0.3035    0.2258   -0.4314
  H2E    H  1.0000    -0.2051    0.0735    0.3867
  H3A    H  1.0000    -0.3095    0.2452   -0.0724
  H3E    H  1.0000    -0.1581    0.4577    0.1744
  H4A    H  1.0000    -0.5000   -0.4321    0.3365
  H4E    H  1.0000    -0.5000   -0.4104    0.0368
```
